# Profiling of polar ionogenic metabolites in Polish wines by capillary electrophoresis‐mass spectrometry

**DOI:** 10.1002/elps.202200066

**Published:** 2022-05-24

**Authors:** Marlien van Mever, Magdalena Fabjanowicz, Maricruz Mamani‐Huanca, Ángeles López‐Gonzálvez, Justyna Płotka‐Wasylka, Rawi Ramautar

**Affiliations:** ^1^ Leiden Academic Centre for Drug Research Leiden University Leiden The Netherlands; ^2^ Department of Analytical Chemistry Faculty of Chemistry Gdańsk University of Technology Gdańsk Poland; ^3^ Centro de Metabolómica y Bioanálisis (CEMBIO) Facultad de Farmacia Universidad San Pablo‐CEU CEU Universities Boadilla del Monte Spain; ^4^ Department of Analytical Chemistry Chemical Faculty and BioTechMed Center Gdańsk University of Technology Gdańsk Poland

**Keywords:** capillary electrophoresis, mass spectrometry, metabolomics, Polish wines

## Abstract

The composition of wine is determined by a complex interaction between environmental factors, genetic factors (i.e., grape varieties), and winemaking practices (including technology and storage). Metabolomics using NMR spectroscopy, GC‐MS, and/or LC‐MS has shown to be a useful approach for assessing the origin, authenticity, and quality of various wines. Nonetheless, the use of additional analytical techniques with complementary separation mechanisms may aid in the deeper understanding of wine's metabolic processes. In this study, we demonstrate that CE‐MS is a very suitable approach for the efficient profiling of polar ionogenic metabolites in wines. Without using any sample preparation or derivatization, wine was analyzed using a 10‐min CE‐MS workflow with interday RSD values for 31 polar and charged metabolites below 3.8% and 23% for migration times and peak areas, respectively. The utility of this workflow for the global profiling of polar ionogenic metabolites in wine was evaluated by analyzing different cool‐climate Polish wine samples.

**Abbreviations**
MVDA: multivariate data analysis
QC: quality control
SIL: stable‐isotope‐labeled
UVDA: univariate data analysis

Grapevine (*Vitis vinifera*) is one of the most widely cultivated fruit crops in the world, which is used to produce juice, dried fruit, and wine [1]. Wine is characterized by a complex matrix in which compounds of distinct structures and belonging to several classes can be found, such as organic acids, polyphenols, vitamins, tannins, anthocyanins, amino acids, and biogenic amines. Metabolomics is a powerful tool providing a holistic view of the unique chemical composition of small molecules (≤1500 Da) in (biological) samples resulting from metabolic processes. This approach has gained momentum for the evaluation of food quality, toxicology, and processing over the past decades [2, 3], and already has a wide application in viticulture in order to evaluate the quality, authenticity [4], factors affecting the sensory characteristic of wine, and terroir effect [5].

In Poland, the most commonly cultivated grapes are hybrid grapevine species that resulted from the crossing of European grapevines (*Vitis vinifera*) with North American grapevines (such as *Vitis rupestis, Vitis riparia* or *Vitis abrusca*) or Asian grapevines (such as *Vitis amurensis*). Crossing two or even more species of *Vitis* resulted in hybrid species of so‐called cold‐climate wines, which are resistant to temperatures below –30°C and also less sensitive to fungal diseases. Specific characteristic features of Polish wines include a higher acidity and lower sugar content as compared to wines from warmer regions [6].

So far, various analytical tools have been developed for metabolomics analysis of grapes and wines [7], in particular GC‐MS, LC‐MS, and NMR have been considered for this purpose. CE‐MS is an analytical approach that offers excellent selectivity for resolving a wide range of polar and charged metabolites as compared to reversed‐phase LC or hydrophilic interaction chromatography (HILIC) [8, 9]. The latter often suffers from a relatively poor retention time reproducibility, complex separation mechanisms, and long equilibration times of columns. The potential of CE‐MS for the analysis of polar ionogenic metabolites in wine has only been reported in a limited number of studies so far [10, 11, 12, 13]. Here, we propose a CE‐MS‐based analytical workflow for the untargeted profiling of polar and charged metabolites in wine and show the utility of this approach by comparative metabolic profiling of cool climate wines from Poland.

For the CE‐MS methodology, we refer to our procedures described previously [14]. Briefly, CE‐MS experiments were carried out on a 7100 CE system hyphenated with a 6230 TOF, both from Agilent Technologies. CE‐MS coupling was realized via a co‐axial sheath‐liquid ESI interface equipped with a triple‐tube sprayer, and sheath‐liquid was of a mixture of water and isopropanol (50:50, v/v) with 0.03% acetic acid, which was delivered at 3 µl/min. As background electrolyte (BGE), 10% (v/v) acetic acid was used. Stable‐isotope‐labeled (SIL) histamine (5 µg/ml) was used as internal standard. CE‐MS experimental data were acquired in positive ionization mode, between 50 and 1000 *m*/*z* with an acquisition rate of 1.5 spectra/s. The following MS settings were used; nebulizer gas: 0 psi, sheath gas (nitrogen) flow rate: 11 L/min, sheath gas temperature: 100°C, ESI capillary voltage: 5500 V, fragmentor voltage: 100 V, skimmer voltage: 50 V. When in‐source fragmentation was required for identification purposes, a fragmentor voltage of 200 V was used. Data treatment and analysis was performed as described previously [15]. Wine samples were purchased from Polish wine stores and included 10 red and 10 white wine samples (see Table S1). Wine samples were ultrasonicated and filtered by a 0.45 µM cellulose filter and stored under dark conditions at room temperature (21°C).

Untargeted metabolomics of wine is a powerful tool for the assessment of wine authenticity and quality. However, before such an approach can be used for this purpose, it is important to assess the performance of CE‐MS first for the analysis of target compounds. Only with acceptable performance metrics (i.e., for repeatability: area RSD% < 25%, migration time RSD% < 10%, linear response function and representable LOD values) obtained for targeted analysis the CE‐MS method can be used for untargeted profiling of metabolites in wine. The analytical performance was evaluated using a metabolite mixture composed of 32 metabolites by considering aspects such as repeatability, response function, and LODs (Tables S2 and S3).

A linear response (and with *R* ^2 ^> 0.981) for the target metabolites in the concentration range from 0.05 to 10 µM was obtained with LODs ranging from 0.002 to 0.218 µg/ml (Table S4). In comparison with previous studies reporting the use of CE‐MS for analysis of metabolites in wine, LODs were at least 2 to 12 times lower for biogenic amines (except for cadaverine) [10, 13]. This improvement is probably due to the use of different CE‐MS separation conditions, such as BGE composition and a lower sheath‐liquid flow rate as no nebulizer gas was applied [9]. Repeatability of the CE‐MS method for direct profiling of metabolites was assessed based on the consecutive analyses of wine samples spiked with metabolite standards (2.5 µg/ml). Intra‐ and interday RSD values for peak areas of all analytes were below 17% (*n* = 5) and 23% (*n* = 15) (except for cadaverine), respectively (Table S4). Migration time repeatability was assessed without internal standard correction, and was below 1.7% and 2.5% for intra‐ and interday analysis, respectively. Given that wine samples were directly analyzed by CE‐MS, the obtained figures of merits for repeatability could be considered acceptable for comparative metabolic profiling studies.

Figure 1 illustrates the applicability of CE‐MS for the direct analysis of biogenic amines and amino acids in pooled red and white wines, respectively. These compound classes could be analyzed within 10 min by using an additional pressure of 40 mbar at the CE inlet during separation, thereby still maintaining a partial separation for the isobaric isomers isoleucine and leucine (*R* = 0.5). In case a better separation would be required, then the use of an additional pressure could be omitted and/or a longer separation capillary could be used.

**FIGURE 1:**
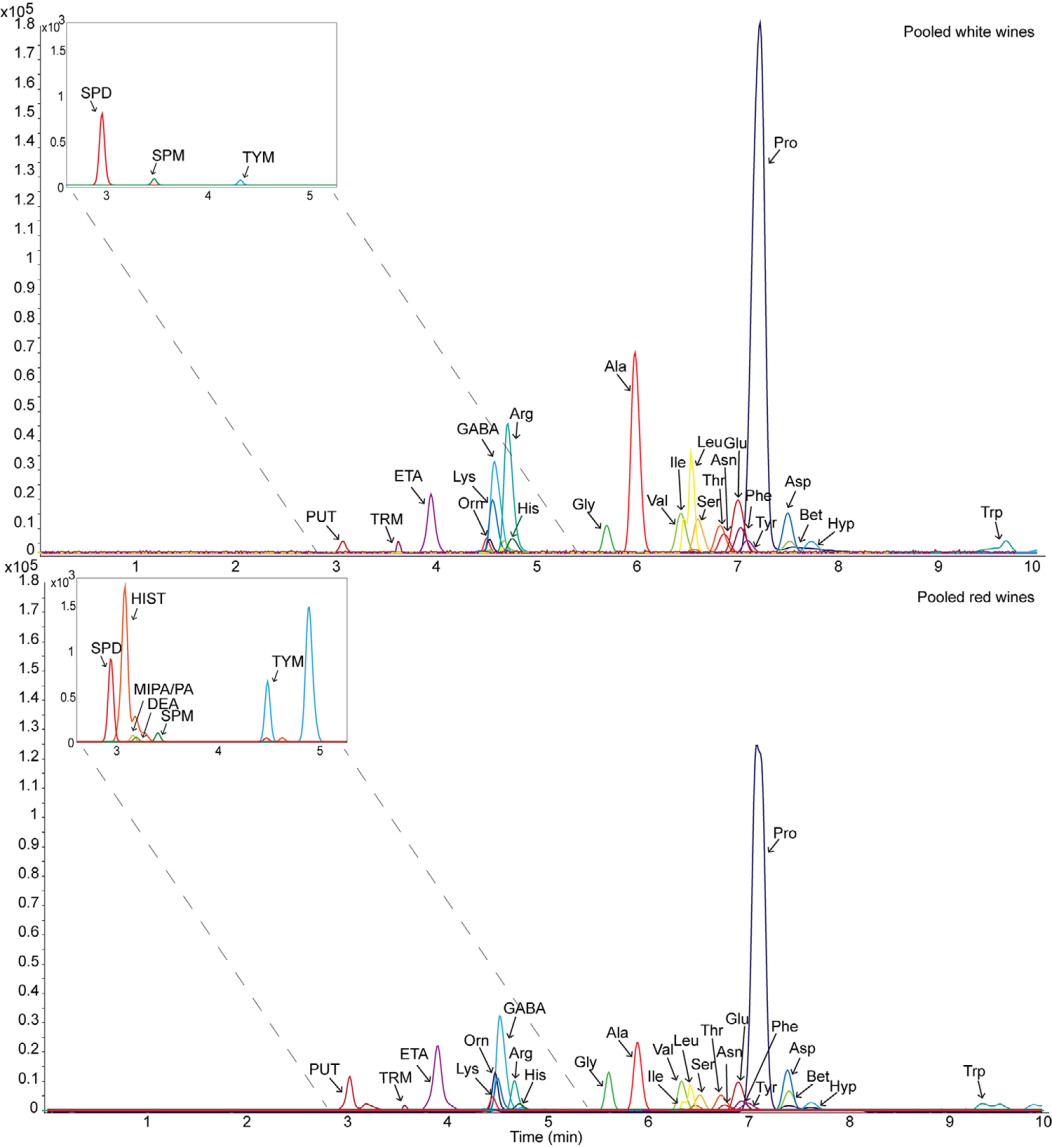
Extracted‐ion electropherograms obtained by CE‐MS for the targeted analysis of (top) pooled white wine and (bottom) pooled red wine. Separation conditions: BGE, 10% acetic acid; sample injection volume 27.4 nl; separation voltage: 30 kV

Matrix effects were assessed using the standard addition method [16]. SIL standards were spiked into pooled wine at concentrations ranging from 0.05 to 25 µg/ml, and resulting response curves were compared to the accompanying response curves obtained when the SIL standards were dissolved in BGE (Figure S5). The slopes were different for all compounds (up to 45% lower for wine samples), indicating that all metabolites experienced a matrix effect. Therefore, for quantitative studies, calibration curves constructed in wine or a SIL internal standard for each compound needs to be used to account for matrix effects.

Next, the CE‐MS workflow was used for untargeted profiling of polar ionogenic metabolites in two groups of samples, that is, red wine (*n* = 10) and white wine (*n* = 10). Quality control (QC) samples (*n* = 5) were prepared by pooling all the wine samples and were analyzed periodically along the sequence to evaluate the performance of the method. A total of 94 features were detected (Table 1), after removing noise signals, duplicates, adducts and fragments. Data quality was assessed by clustering QC samples measurements in an unsupervised PCA‐X model. The model showed a *R* ^2 ^= 0.735, which indicated the high quality of the analysis (Figure 2) and the clustering trends of the groups were observed.

**TABLE 1:** Tentative identification of metabolites observed in pooled Polish wine samples by CE‐MS after data treatment

Compound name	*m/z*	μ_eff_ in wines	Formula	Level ID	In‐source fragments at 200 V
Glycine	76.0396	1213.0	C_2_H_5_NO_2_	L1	
Alanine	90.0549	1079.5	C_3_H_7_NO_2_	L1	
Serine	106.0492	828.2	C_3_H_7_NO_3_	L1	60.0450, 88.0393
Proline	116.0704	586.1	C_5_H_9_NO_2_	L1	
Valine	118.0855	895.2	C_5_H_11_NO_2_	L1	
Betaine	118.0856	522.9	C_5_H_11_NO_2_	L1	
Threonine	120.0650	747.6	C_4_H_9_NO_3_	L1	74.0614, 102.0561
Pipecolic acid	130.0855	810.2	C_6_H_11_NO_2_	L3	
Isoleucine/leucine	132.0998	872.5	C_6_H_13_NO_2_	L1	69.0826, 86.0982
Asparagine	133.0595	740.8	C_4_H_8_N_2_O_3_	L1	70.0317, 74.0241, 116.0366
Aspartic acid	134.0441	536.9	C_4_H_7_NO_4_	L1	70.0302, 74.0254, 88.0409
Glutamine	147.0759	730.9	C_5_H_10_N_2_O_3_	L3	
Lysine	147.1121	1907.9	C_6_H_14_N_2_O_2_	L1	84.0826, 102.0947, 130.0877
Glutamic acid	148.0594	694.5	C_5_H_9_NO_4_	L1	84.0459, 102.0556, 130.0506
Methionine	150.0581	751.0	C_5_H_11_NO_2_S	L1	
Histidine	156.0761	1778.9	C_6_H_9_N_3_O_2_	L1	83.0619,110.0725
O‐Acetylhomoserine/aminoadipic acid	162.0755	568.5	C_6_H_11_NO_4_	L3	
Phenylalanine	166.0854	691.2	C_9_H_11_NO_2_	L1	120.0812, 131.0429, 149.0636
Arginine	175.1187	1792.8	C_6_H_14_N_4_O_2_	L1	60.0575, 70.0669, 116.0722, 158.0944
Citrulline	176.1003	704.3	C_6_H_13_N_3_O_3_	L3	
Tyrosine	182.0805	665.6	C_9_H_11_NO_3_	L1	123.0422, 136.0737, 147.0419, 165.0525
Cytidine	244.0928	1272.1	C_9_H_13_N_3_O_5_	L1	
Nicotianamine	304.1494	444.8	C_12_H_21_N_3_O_6_	L3	
**Biogenic amines**					
Ethanolamine	62.0608	2486.7	C_2_H_7_NO	L1	
Putrescine	89.1073	3690.6	C_4_H_12_N_2_	L1	
Beta‐alanine	90.0551	1968.4	C_3_H_7_NO_2_	L1	
GABA	104.0701	1976.8	C_4_H_9_NO_2_	L1	
Tyramine	138.0915	1652.5	C_8_H_11_NO	L1	91.0536, 103.0531, 105.0442, 121.065
**Amino acids and derivatives**					
Pyroglutamine/Dihydrothymine	129.0653	1247.2	C_5_H_8_N_2_O_2_	L3	
4‐Hydroxyproline	132.0650	479.1	C_5_H_9_NO_3_	L1	68.0506, 86.0615, 114.0534
3‐Aminocaproic acid	132.1003	1820.9	C_6_H_13_NO_2_	L3	
*Cis*‐4‐(Hydroxymethyl)‐2‐pyrrolidinecarboxylate	146.0804	734.1	C_6_H_11_NO_3_	L2	82.0664,100.0765,128.0713
8/3/2‐Aminooctanoic acid	160.1327	1476.4	C_8_H_17_NO_2_	L3	
*N*‐Acetyl‐2,4‐diaminobutanoate/Ala‐Ala	161.0946	1338.9	C_6_H_12_N_2_O_3_	L3	
Methionine sulfoxide/ethiin	166.0544	534.1	C_5_H_11_NO_3_S	L3	
*N*_2_‐Acetyl‐ornithine/theanine	175.1068	1257.1	C_7_H_14_N_2_O_3_	L2	129.0657
Ethyl glutamate/2‐aminoheptanedioic acid/hydroxyvalerylglycine	176.0917	1403.4	C_7_H_13_NO_4_	L3	
**Amino acids and derivatives**					
Ethyl glutamate/2‐aminoheptanedioic acid/hydroxyvalerylglycine	176.0925	691.2	C_7_H_13_NO_4_	L3	
*N*‐Hydroxy‐phenylalanine/meta‐tyrosine	182.0809	595.0	C_9_H_11_NO_3_	L3	
Homoarginine/targinine	189.1339	1678.3	C_7_H_16_N_4_O_2_	L3	
4‐(Glutamylamino) butanoate/*N*_2_‐succinyl‐ornithine/Asp‐Val	233.1133	1019.4	C_9_H_16_N_2_O_5_	L3	
γ‐Glutamyl‐pipecolic acid/(2S,2′S)‐pyrosaccharopine	259.1280	308.8	C_11_H_18_N_2_O_5_	L3	
Cyclic argininosuccinic acid derivative 1	273.1189	1338.9	C_10_H_16_N_4_O_5_	L2	70.0645
*N*_6_‐(Octanoyl)lysine	273.2158	1124.1	C_14_H_28_N_2_O_3_	L3	
*N*_2_‐Fructopyranosylarginine	337.1700	1165.5	C_12_H_24_N_4_O_7_	L3	
**Peptides** ^a^					
Proline betaine	144.1030	1772.0	C_7_H_13_NO_2_	L2	72.0822, 84.0820
Ala Ser	177.0860	1262.1	C_6_H_12_N_2_O_4_	L3	
Pro‐Ala	187.1071	1282.2	C_8_H_14_N_2_O_3_	L3	
Gly Leu	189.1219	1213.0	C_8_H_16_N_2_O_3_	L3	
Ala‐Thr	191.1010	1208.2	C_7_H_14_N_2_O_4_	L3	
Leu‐Ala	203.1380	1179.6	C_9_H_18_N_2_O_3_	L3	86.0978
Thr‐Ser	207.0940	1137.8	C_7_H_14_N_2_O_5_	L3	
Valyl‐Betaine	217.1527	1142.4	C_10_H_20_N_2_O_3_	L3	
Val Val	217.1528	1282.2	C_10_H_20_N_2_O_3_	L3	
Thr‐Val/Ser‐Leu	219.1308	1133.2	C_9_H_18_N_2_O_4_	L3	
Asp‐Ser	221.0800	3057.5	C_7_H_12_N_2_O_6_	L3	
Gly‐Phe	223.1088	1137.8	C_11_H_14_N_2_O_3_	L1	
Val‐Ile	231.1691	1106.1	C_11_H_22_N_2_O_3_	L3	
Ile‐Val	231.1695	1106.1	C_11_H_22_N_2_O_3_	L3	
Leu‐Thr	233.1470	1088.3	C_10_H_20_N_2_O_4_	L3	86.0982
Ile‐Ile	245.1844	1079.5	C_12_H_24_N_2_O_3_	L3	86.0982
Asp‐Ile/Glu‐Val	247.1253	978.0	C_10_H_18_N_2_O_5_	L3	
Asp‐Ile/Glu‐Val	247.1266	978.0	C_10_H_18_N_2_O_5_	L3	
Leu‐Lys	260.1954	1991.6	C_12_H_25_N_3_O_3_	L3	
Glu‐Leu	261.1448	1011.0	C_11_H_20_N_2_O_5_	L3	
Glu Lys	276.1532	1040.6	C_11_H_21_N_3_O_5_	L3	
Val Gly Leu	288.1903	1023.6	C_13_H_25_N_3_O_4_	L3	
Ile‐Arg	288.2028	1968.4	C_12_H_25_N_5_O_3_	L3	
Gly Thr Leu	290.1701	1006.8	C_12_H_23_N_3_O_5_	L3	
Asp Cys Gly	294.0712	2834.8	C_9_H_15_N_3_O_6_S	L3	
Leu Ala Val	302.2053	990.3	C_14_H_27_N_3_O_4_	L3	86.0979
Other compounds					
Choline	104.1069	2206.0	C_5_H_14_NO	L2	60.0816
Picolinic acid/nicotinic acid	124.0394	751.0	C_6_H_5_NO_2_	L3	
Imidazoleacetic acid/thymine	127.0495	1646.1	C_5_H_6_N_2_O_2_	L3	
Adenine	136.0621	1820.9	C_5_H_5_N_5_	L3	
Hypoxanthine	137.0449	457.9	C_5_H_4_N_4_O	L3	
**Other compounds**					
Trigonelline	138.0539	678.4	C_7_H_7_NO_2_	L3	
Imidazolelactic acid	157.0602	1370.8	C_6_H_8_N_2_O_3_	L2	111.0568
3‐Dehydroxycarnitine	146.1171	1583.4	C_7_H_15_NO_2_	L3	
Carnitine	162.1123	1546.9	C_7_H_15_NO_3_	L3	
Ethyl *N*‐ethylanthranilate	194.1148	1408.9	C_11_H_15_NO_2_	L3	
MTCA	231.1117	465.8	C_13_H_14_N_2_O_2_	L2	158.0957, 214.0859
Glycylprolylhydroxyproline	286.1382	846.5	C_12_H_19_N_3_O_5_	L3	
**Unknown compounds**					
	108.0654	1092.7		L4	
	138.0520	659.3		L4	
	139.6059	2129.0		L4	
	158.1249	1608.2		L4	
	160.6280	1991.6		L4	
	231.1116	465.8		L4	
	250.1750	1930.4		L4	
	263.1118	2980.9		L4	
	274.2694	1083.9		L4	
	285.0767	3110.0		L4	
	345.0877	3006.1		L4	

^a^Contains multiple identification options.

Abbreviation: MTCA, (1xi,3xi)‐1,2,3,4‐tetrahydro‐1‐methyl‐beta‐carboline‐3‐carboxylic acid.

**FIGURE 2:**
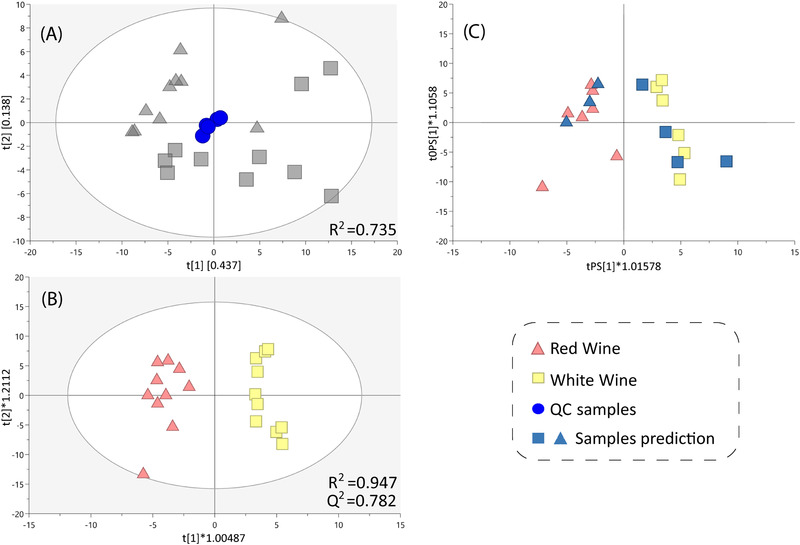
SIMCA‐P software (Version 17, Umetrics, Sartorius Stedim Biotech) was used to perform multivariate analysis models. (A) Principal component analysis (PCA‐X) score plot with an explained variance *R* ^2 ^= 0.735, using non‐normalized samples wines (gray color) and quality control (QC, blue circle). (B) Orthogonal partial least‐squares‐discriminant analysis (OPLS‐DA) analysis of variation between red wine and white wine samples (*R* ^2^ = 0.947, *Q* ^2^ = 0.782) and CV‐ANOVA *p*‐value = 1.06 × 10^–03^. (C) Plot corresponding to the cross‐validation for the OPLS‐DA model

The compounds that play the most significant role in the discrimination between wines of different types and origin are amino acids. Key amino acids include proline, which is the most abundant amino acid in grapes. Its level is determined by the grape variety and aromatic amino acid phenylalanine, whose level depends on grape variety and alcoholic fermentation, where it is used by bacteria, fungi, and yeast to produce a highly polar aromatic alcohol, phenethyl alcohol [17]. A supervised Orthogonal partial least‐squares‐discriminant analysis model was built to evaluate the metabolic differences between white and red wine (Figure 2). The model showed a high grade of discrimination between the groups of samples and good quality parameters (*R* ^2 ^= 0.947, *Q* ^2 ^= 0.782). The model was validated by CV‐ANOVA (*p*‐value = 1.06 × 10^–03^) and by the cross‐validation leaving 1/3 out approach, showing a prediction accuracy of 100% (Figure 2). In order to identify the features that were statistically significant by multivariate data analysis (MVDA) analysis, the confidence intervals of Jack‐Knife, correlation *p*(_corr_) > |0.5|, and variables importance in projection (VIP) > 1 were calculated. Additionally, the univariate data analysis (UVDA) statistical analysis was performed in order to obtain the statistical significance of each compound in the comparison of both groups. Metabolites with *p *< 0.05 were selected as significant metabolites using the Mann–Whitney *U* test; for correction of comparisons, the Benjamini–Hochberg method was applied to all *p*‐values to control the false discovery rate (FDR) at the *q* = 0.05 level. The overall statistical analysis revealed 45 metabolic features as significantly different between the groups (Table S6). The annotation of these features was performed by *m/z* search in the FooDB database and CMM, a search tool that integrates different databases (Kegg, HMDB, LipidMaps, METLIN, NPAtlas, KNApSAcK, MINE) and an in‐house library [18]. The annotation was carried out considering mass accuracy (20 ppm as maximum error as recommended [19]), electrophoretic mobility (<5% error to effective mobility database [9, 20]), isotopic pattern, and adduct formation (confidence level 3). This was considered as tentative annotation. Additionally, in order to increase this confidence level, in‐source fragmentation was performed (confidence level 2) [15], and when available, some metabolites were identified using commercial standards (confidence level 1). For unknown metabolites, only *m/z* was considered (confidence level 4).

Interestingly, cyclic argininosuccinic acid was found to be one of the metabolites responsible for the classification of the two sets of wine samples, expressed more in white wines. This compound is found in a dynamic equilibrium with argininosuccinic acid in its open form; at an acidic pH as is the case in wines, cyclic argininosuccinic acid predominates [21].

Overall, a CE‐MS workflow for the direct profiling of polar ionogenic metabolites in wine is proposed and a proof‐of‐principle study utilizing white and red cool‐climate wines originating from Poland revealed the potential of this approach for assessing wine authenticity and quality in follow‐up studies.

## CONFLICT OF INTEREST

The authors have declared no conflict of interest.

## Supporting information

Supporting information

## Data Availability

The data that support the findings of this study are available from the corresponding author upon reasonable request.
