# Supplementary material for: Profiling of polar ionogenic metabolites in Polish wines by capillary electrophoresis‐mass spectrometry
Source: Electrophoresis. 2022 May 24;43(18-19):1814–21. doi: 10.1002/elps.202200066 (PMC9790660; doi:10.1002/elps.202200066)
Supplement: Supplementary file 1 — Supporting information [file ELPS-43-1814-s001.docx]

**(Supplementary Information)**

**Profiling polar ionogenic metabolites in Polish wines by CE-MS**

Marlien van Mever^1*^, Magdalena Fabjanowicz^2*^, Maricruz Mamani-Huanca^3^, Ángeles López-Gonzálvez^3^, Justyna Płotka-Wasylka^4#^, Rawi Ramautar^1#^

^1^Leiden Academic Centre for Drug Research, Leiden University, Leiden, The Netherlands

^2^ Department of Analytical Chemistry, Faculty of Chemistry, Gdańsk University of Technology, Gdańsk, Poland

^3^ Centro de Metabolómica y Bioanálisis (CEMBIO), Facultad de Farmacia, Universidad San Pablo-CEU, CEU Universities, Urbanización Montepríncipe, 28660 Boadilla del Monte, Spain

^4^ Department of Analytical Chemistry, Chemical Faculty and BioTechMed Center, Gdańsk University of Technology, Poland

*Corresponding author: Marlien van Mever,

Leiden Academic Centre for Drug Research (LACDR),

Leiden University, 2333 CC Leiden, The Netherlands

E-mail: [m.van.mever@lacdr.leidenuniv.nl](mailto:m.van.mever@lacdr.leidenuniv.nl)

Magdalena Fabjanowicz,

Department of Analytical Chemistry,

Gdańsk University of Technology, 80-233 Gdańsk, Poland

E-mail: [magfabja@student.pg.edu.pl](mailto:magfabja@student.pg.edu.pl)

**Table S-1**. Information about the Polish wines.

| **Label** | **Year** | **Type of wine** | **Origin** | **% Alcohol** | **Grape type** | **Sugar content** |
| --- | --- | --- | --- | --- | --- | --- |
| 1R | 2015 | Red | Paczkow | 11 | Regent | dry |
| 2R | 2017 | Red | Grybów | 12 | Regent | dry |
| 3R | 2014 | Red | Bliskowice | 12.1 | Rondo | dry |
| 4R | 2013 | Red | Witanowice | 12.5 | Regent | dry |
| 5R | 2017 | Red | Zachowice | 13.5 | Dornfelder | dry |
| 6R | 2017 | Red | Grybów | 11 | Rondo | dry |
| 7R | 2017 | Red | Zachowice | 13.5 | Pinot Noir | dry |
| 8R | 2016 | Red | Banie | 13 | Rondo/Regent | dry |
| 9R | 2015 | Red | Paczków | 11.5 | Rondo | dry |
| 10R | 2016 | Red | Jasło | 12.5 | Mix of 3 grapes | dry |
| 1W | 2016 | White | Opole Lubelskie | 12 | Johanniter | dry |
| 2W | 2017 | White | Zachowice | 12 | Riesling | semi-dry |
| 3W | 2016 | White | Borów Wielki | 12 | Pinot Gris, Riesling, Muscat Ottonel, Gewurztraminer | semi-dry |
| 4W | 2017 | White | Kraków | 12 | Seyval Blanc, Hibernal, Johanniter, Solaris | semi-dry |
| 5W | 2016 | White | Borów Wielki | 13 | Pinot Gris | semi-dry |
| 6W | 2016 | White | Opole Lubelskie | 12.5 | Solaris | sweet |
| 7W | 2014 | White | Witanowice | 12 | Bianca | dry |
| 8W | 2017 | White | Banie | 12.5 | Solaris | dry |
| 9W | 2017 | White | Jasło | 12 | Mix of grapes | semi-sweet |
| 10W | 2015 | White | Jaslo | 11.5 | Mix of 8 grapes | dry |

**Table S-2.** Standard metabolites and suppliers list.

| Name | Formula | Mass | Commercial | CAS Nº |
| --- | --- | --- | --- | --- |
| Isopropylamine | C_3_H_9_N | 59.0735 | Fluka | 75-31-0 |
| Propylamine | C_3_H_9_N | 59.0735 | Fisher Scientific | 107-10-8 |
| Ethanolamine | C_2_H_7_NO | 61.0528 | Sigma Aldrich | 141-43-5 |
| Diethylamine | C_4_H_11_N | 73.0891 | Fisher Scientific | 109-89-7 |
| Glycine | C_2_H_5_NO_2_ | 75.0320 | Sigma Aldrich | 56-40-6 |
| Alanine | C_3_H_7_NO_2_ | 89.0477 | Sigma Aldrich | 56-41-7 |
| Gamma-aminobutyric acid | C_4_H_9_NO_2_ | 103.0633 | Sigma Aldrich | 56-12-2 |
| Choline | C_5_H_14_NO | 104.1075 | Sigma Aldrich | 67-48-1 |
| Serine | C_3_H_7_NO_3_ | 105.0426 | Sigma Aldrich | 56-45-1 |
| Histamine | C_5_H_9_N_3_ | 111.0796 | Sigma Aldrich | 51-45-6 |
| Proline | C_5_H_9_NO_2_ | 115.0633 | Sigma Aldrich | 147-85-3 |
| Valine | C_5_H_11_NO_2_ | 117.0790 | Sigma Aldrich | 72-18-4 |
| Betaine | C_5_H_12_NO_2_ | 118.0868 | Sigma Aldrich | 6915-17-9 |
| Putrescine | C_3_H_5_NO_4_ | 119.0219 | Sigma Aldrich | 1068-84-4 |
| Spermidine | C_4_H_9_NO_3_ | 119.0582 | Sigma Aldrich | 28954-12-3 |
| Threonine | C_4_H_9_NO_3_ | 119.0582 | Sigma Aldrich | 72-19-5 |
| *Trans*-4-hydroxyproline | C_5_H_9_NO_3_ | 131.0582 | Sigma Aldrich | 51-35-4 |
| Leucine | C_6_H_13_NO_2_ | 131.0946 | Sigma Aldrich | 61-90-5 |
| Isoleucine | C_6_H_13_NO_2_ | 131.0946 | Sigma Aldrich | 73-32-5 |
| Asparagine | C_4_H_8_N_2_O_3_ | 132.0535 | Sigma Aldrich | 70-47-3 |
| Aspartic acid | C_4_H_7_NO_4_ | 133.0375 | Sigma Aldrich | 56-84-8 |
| Tyramine | C_8_H_11_NO | 137.0841 | Sigma Aldrich | 51-67-2 |
| Glutamine | C_5_H_10_N_2_O_3_ | 146.0691 | Sigma Aldrich | 56-85-9 |
| Lysine | C_6_H_14_N_2_O_2_ | 146.1055 | Sigma Aldrich | 56-87-1 |
| Glutamic acid | C_5_H_9_NO_4_ | 147.0532 | Sigma Aldrich | 56-86-0 |
| Methionine | C_5_H_11_NO_2_S | 149.0510 | Sigma Aldrich | 63-68-3 |
| Paracetamol | C_8_H_9_NO_2_ | 151.0633 | Fluka | 103-90-2 |
| Histidine | C_6_H_9_N_3_O_2_ | 155.0695 | Sigma Aldrich | 71-00-1 |
| Tryptamine | C_10_H_12_N_2_ | 160.1000 | Sigma Aldrich | 61-54-1 |
| Phenylalanine | C_9_H_11_NO_2_ | 165.0790 | Sigma Aldrich | 63-91-2 |
| Arginine | C_6_H_14_N_4_O_2_ | 174.1117 | Sigma Aldrich | 74-79-3 |
| Tyrosine | C_9_H_11_NO_3_ | 181.0740 | Sigma Aldrich | 60-18-4 |
| Tryptophan | C_11_H_12_N_2_O_2_ | 204.0899 | Sigma Aldrich | 73-22-3 |
| Gly-phe | C_11_H_14_N_2_O_3_ | 222.1004 | Sigma Aldrich | 3321-03-7 |
| Procaine | C_13_H_20_N_2_O_2_ | 236.1525 | Sigma Aldrich | 51-05-8 |

**Table S-2 Cont.** Standard compounds and suppliers list.

| Name Compound | Formula | Mass | Commercial | CAS Nº |
| --- | --- | --- | --- | --- |
| Argininosuccinic acid | C_10_H_18_N_4_O_6_ | 290.1226 | Merck | 918149-29-8 |
| Adonitol | C_5_H_12_O_5_ | 152.0684 | Sigma Aldrich | 488-81-3 |
| Choline-chloride | (CH_3_)_3_N(Cl)CH_2_CH_2_OH | 139.0764 | Sigma Aldrich | 67-48-1 |
| L-aspartic acid-13C4,15N | HOOCCH_2_CH(NH_2_)COOH | 141.0375 | Cambridge Isotope Laboratories | 202468-27-7 |
| L-glutamine-13C | H_2_NCO(CH_2_)_2_CH(NH_2_)COOH | 148.0691 | Cambridge Isotope Laboratories | 159663-16-8 |
| Histamine-d4 | C_5_D_4_H5N_3_ | 115.1047 | Cambridge Isotope Laboratories | 344299-48-5 |
| L-lysine-13C | H_2_N(CH_2_)_4_CH(NH_2_)COOH | 152.1055 | Cambridge Isotope Laboratories | 202190-50-9 |
| L-asparagine-13C,15N | H_2_NCOCH_2_CH(NH_2_)COOH | 135.0534 | Cambridge Isotope Laboratories | 202406-87-9 |
| L-isoleucine-13C,15N | CH_3_CH_2_CH(CH_3_)CH(NH_2_)COOH | 133.0946 | Cambridge Isotope Laboratories | 202406-52-8 |
| L-glutamic acid-13C,15N | HOOC(CH_2_)_2_CH(NH_2_)COOH | 158.0531 | Cambridge Isotope Laboratories | NA |
| L-glutamine-13C | H_2_NCO(CH_2_)_2_CH(NH_2_)COOH | 148.0691 | Cambridge Isotope Laboratories | 184161-19-1 |
| Spermine-d8 | C_10_D_8_H_18_N_4_ | 153.2081 | Sigma Aldrich | 1173022-85-9 |

**Table S-3*.*** An overview of physico-chemical properties of the metabolites.

| Compound | Average MW (g/mol) | Monoisotopic MW | *m/z* | pKa (strongest acidic) | pKa (strongest basic) | Log D^a^ |
| --- | --- | --- | --- | --- | --- | --- |
| Isopropylamine | 59.11 | 59.0735 | 60.0808 | n/a | 10.43 | 0.26 |
| Propylamine | 59.11 | 59.0735 | 60.0808 | n/a | 10.21 | 0.48 |
| Ethanolamine | 61.08 | 61.0528 | 62.0601 | 15.61 | 9.55 | -1.30 |
| Diethylamine | 73.14 | 73.0891 | 74.0964 | n/a | 10.58 | 0.52 |
| Glycine | 75.07 | 75.0320 | 76.0393 | 2.37 | 9.24 | -3.21 |
| Putrescine | 88.15 | 88.1000 | 89.1073 | n/a | 10.51 | -0.85 |
| Alanine | 89.09 | 89.0477 | 90.0550 | 2.47 | 9.48 | -2.85 |
| Cadaverine | 102.18 | 102.1157 | 103.1229 | n/a | 10.51 | -0.40 |
| Gamma-aminobutyric acid | 103.12 | 103.0633 | 104.0706 | 4.53 | 10.2 | -3.17 |
| Serine | 105.09 | 105.0426 | 106.0499 | 2.03 | 8.93 | -3.07 |
| Histamine | 111.15 | 111.0796 | 112.0869 | 14.46 | 9.58 | -0.70 |
| Proline | 115.13 | 115.0633 | 116.0706 | 1.94 | 11.3 | -2.54 |
| Valine | 117.15 | 117.0790 | 118.0863 | 2.72 | 9.60 | -2.26 |
| Betaine | 118.15 | 117.0790 | 118.0863 | 2.26 | n/a | -4.50 |
| Threonine | 119.12 | 119.0582 | 120.0655 | 2.21 | 9.00 | -2.94 |
| *Trans*-4-hydroxyproline | 131.13 | 131.0582 | 132.0655 | 1.64 | 10.6 | -3.17 |
| Isoleucine | 131.17 | 131.0946 | 132.1019 | 2.79 | 9.59 | -1.70 |
| Leucine | 131.17 | 131.0946 | 132.1019 | 2.79 | 9.52 | -1.52 |
| Asparagine | 132.12 | 132.0535 | 133.0608 | 2.00 | 8.43 | -3.82 |
| Ornithine | 132.16 | 132.0899 | 133.0972 | 2.67 | 10.3 | -4.22 |
| Aspartic acid | 133.10 | 133.0375 | 134.0448 | 1.70 | 9.61 | -3.89 |
| Tyramine | 137.18 | 137.0841 | 138.0913 | 10.41 | 9.66 | 0.68 |
| Spermidine | 145.25 | 145.1579 | 146.1652 | n/a | 10.68 | -1.10 |
| Lysine | 146.19 | 146.1055 | 147.1128 | 2.74 | 10.3 | -3.05 |
| Glutamic acid | 147.13 | 147.0532 | 148.0604 | 1.88 | 9.54 | -3.69 |
| Histidine | 155.15 | 155.0695 | 156.0768 | 1.85 | 9.44 | -3.32 |
| Tryptamine | 160.22 | 160.1000 | 161.1073 | 17.17 | 9.73 | 1.49 |
| Phenylalanine | 165.19 | 165.0790 | 166.0863 | 2.47 | 9.45 | -1.38 |
| Arginine | 174.20 | 174.1117 | 175.1190 | 2.41 | 12.4 | -4.20 |
| Tyrosine | 181.19 | 181.0739 | 182.0812 | 2.00 | 9.19 | -2.26 |
| Spermine | 202.34 | 202.2157 | 203.2230 | n/a | 10.8 | -1.50 |
| Tryptophan | 204.23 | 204.0899 | 205.097223 | 2.54 | 9.40 | -1.10 |

Values adapted from https://hmdb.ca , (^a^) D = distribution coefficient, n/a **=** not applicable

**Table S-4**. Analytical performance characteristics obtained for the direct analysis of selected metabolites in wine by CE-MS.

| Compound | Linear range (µg/mL) | R^2^ | LOD (µg/mL) | Peak area repeatability in matrix (RSD%)^a^ | | MT repeatability in matrix (RSD%)^a^ | |
| --- | --- | --- | --- | --- | --- | --- | --- |
|  |  |  |  | Intraday (*n*=5) | Interday (*n*=15) | Intraday (*n*=5) | Interday (*n*=15) |
| Isopropylamine | 0.05 - 10 | 0.998 | 0.032 | 5.8 | 9.1 | 0.8 | 1.0 |
| Propylamine | 0.1 - 10 | 0.987 | 0.002 | 4.1 | 17.7 | 1.3 | 1.1 |
| Ethanolamine | 0.05 - 10 | 0.999 | 0.048 | 6.9 | 15.6 | 0.5 | 1.0 |
| Diethylamine | 0.1 - 10 | 0.997 | 0.11 | 9.5 | 11.1 | 1.3 | 1.8 |
| Putrescine | 0.1 - 10 | 0.999 | 0.014 | 8.8 | 7.5 | 0.6 | 0.7 |
| Histamine | 0.05 - 10 | 0.999 | 0.002 | 5.1 | 10.3 | 0.6 | 0.9 |
| Tyramine | 0.05 - 7.5 | 0.998 | 0.009 | 10.1 | 13.4 | 0.7 | 1.1 |
| Spermine | 0.25 - 10 | 0.999 | 0.016 | 6.6 | 10.0 | 0.4 | 3.8 |
| Tryptamine | 0.05 - 10 | 0.993 | 0.003 | 12.4 | 12.2 | 1.0 | 1.2 |
| Spermidine | 0.05 - 10 | 0.992 | 0.004 | 9.8 | 17.3 | 0.4 | 1.1 |
| Cadaverine | 0.3 - 10 | 0.981 | 0.218 | 20.7 | 34.2 | 0.9 | 2.5 |
| Glycine | 0.2 - 7.5 | 0.999 | 0.14 | 13.0 | 13.3 | 1.2 | 1.5 |
| Alanine | 0.2 - 18 | 0.991 | 0.42 | 13.4 | 11.9 | 0.9 | 1.5 |
| Serine | 0.3 - 21 | 0.998 | 0.29 | 14.4 | 13.8 | 1.3 | 1.7 |
| Proline | 0.6 - 23 | 0.999 | 0.74 | 11.5 | 10.5 | 0.8 | 1.5 |
| Tryptophan | 0.5 - 41 | 0.999 | 1.00 | 15.8 | 22.9 | 1.7 | 2.5 |
| Valine | 0.3 - 23 | 0.995 | 0.23 | 13.2 | 13.1 | 1.1 | 1.5 |
| Betaine | 0.3 - 23 | 0.987 | 0.00 | 9.2 | 7.8 | 1.5 | 1.8 |
| Threonine | 0.06 -24 | 0.995 | 0.07 | 16.8 | 19.5 | 1.4 | 1.6 |
| Leucine and Isoleucine^b^ | 0.3 - 26 | 0.994 | n/a | 12.6 | 11.9 | 0.7 | 1.4 |
| Asparagine | 0.3 - 26 | 0.996 | 0.01 | 15.4 | 14.7 | 1.2 | 1.7 |
| Ornithine | 0.3 - 26 | 0.986 | 0.54 | 11.2 | 11.4 | 1.0 | 1.2 |
| Aspartic acid | 0.3 - 13.5 | 0.999 | 0.05 | 13.6 | 14.7 | 1.1 | 1.8 |
| Lysine | 0.2 - 29 | 0.981 | 0.39 | 7.8 | 9.3 | 1.3 | 2.1 |
| Glutamic acid | 0.4 - 29 | 0.999 | 0.27 | 14.1 | 13.3 | 0.8 | 1.3 |
| Histidine | 0.4 - 15.2 | 0.998 | 0.04 | 10.6 | 9.8 | 1.1 | 1.6 |
| Phenylalanine | 0.4 - 33 | 0.998 | 0.10 | 13.8 | 14.4 | 0.9 | 1.4 |
| Arginine | 0.2 - 35 | 0.997 | 0.78 | 8.9 | 8.8 | 1.2 | 1.5 |
| Tyrosine | 0.2 - 36 | 0.999 | 0.02 | 13.9 | 15.4 | 0.9 | 1.2 |
| Gamma-aminobutyric acid | 0.3 - 21 | 0.996 | 0.60 | 8.9 | 8.3 | 1.3 | 1.7 |
| *Trans*-4-hydroxyproline | 0.2 - 13 | 0.998 | 0.28 | 12.0 | 13.5 | 1.0 | 1.3 |

(^a^) For red wine (4R) spiked with metabolite standards at a concentration of 2.5 µg/mL, (^b^) integrated as one peak, n/a **=** not applicable

**Figure S-5.** Calibration curves for (A) metabolite standards in water and (B) metabolites spiked in pooled wine. The standard concerned is indicated above the figure. Separation conditions: BGE, 10% acetic acid; sample injection volume 27.4nL.


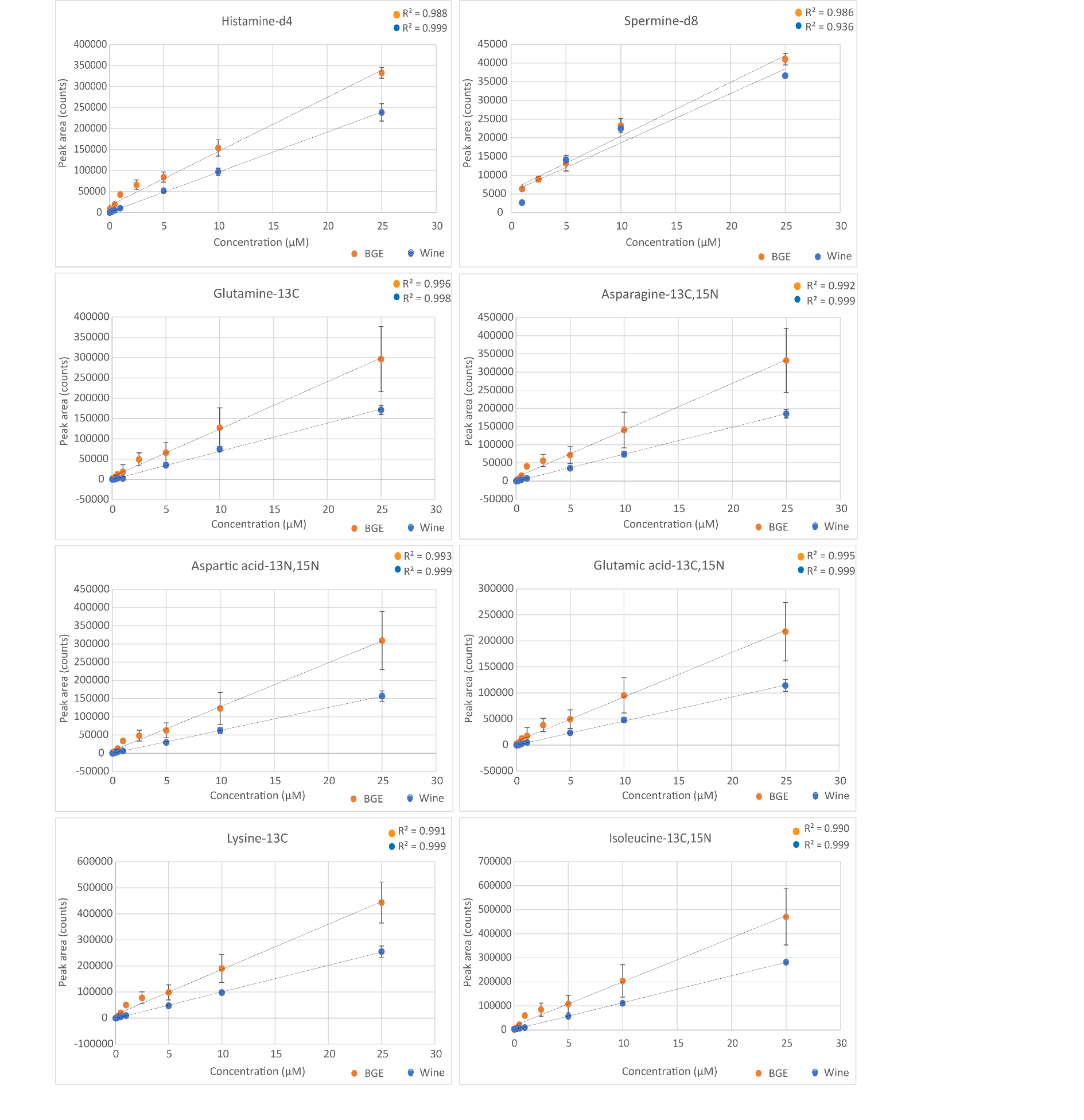


**Table S-6** List containing the statistically significant annotated metabolites discriminating between red and white wine profiles with their statistical characteristics after MVDA and UVDA.

|  |  |  |  |  | **MVDA** | | | **UVDA** | | **Fold change** |
| --- | --- | --- | --- | --- | --- | --- | --- | --- | --- | --- |
| **Compound Name** | **%RSD** | ***m/z*** | **MT**  **(min)** | **Formula** | ***p*(corr)** | **VIP** | **JK(^b^)** | ***p-*value** | **FDR (^c^)** |  |
| Isoleucine/Leucine | 7.8 | 132.0998 | 6.77 | C_6_H_13_NO_2_ | 0.41 | 1.07 | Yes | 1.4E-01 | 1.9E-01 | 1.71 |
| Asparagine | 9.6 | 133.0595 | 7.11 | C_4_H_8_N_2_O_3_ | 0.64 | 1.23 | Yes | 2.8E-03 | 1.9E-02 | 2.66 |
| Lysine | 8.6 | 147.1121 | 4.79 | C_6_H_14_N_2_O_2_ | 0.41 | 1.03 | No | 5.4E-02 | 1.5E-01 | 2.35 |
| Methionine | 7.0 | 150.0581 | 7.14 | C_5_H_11_NO_2_S | 0.76 | 1.44 | Yes | 1.8E-04 | 3.5E-03 | 10.27 |
| Histidine | 7.7 | 156.0761 | 5.00 | C_6_H_9_N_3_O_2_ | 0.63 | 1.26 | Yes | 1.7E-02 | 5.5E-02 | 2.50 |
| O-Acetylhomoserine/ Aminoadipic acid | 11.1 | 162.0755 | 7.67 | C_6_H_11_NO_4_ | 0.46 | 1.06 | Yes | 8.9E-02 | 1.9E-01 | 2.04 |
| Phenylalanine | 5.4 | 166.0854 | 7.28 | C_9_H_11_NO_2_ | 0.51 | 1.15 | Yes | 6.4E-02 | 1.1E-01 | 2.08 |
| Tyrosine | 12.0 | 182.0805 | 7.35 | C_9_H_11_NO_3_ | 0.43 | 1.03 | Yes | 4.5E-02 | 1.5E-01 | 2.14 |
| Nicotianamine | 19.7 | 304.1494 | 8.13 | C_12_H_21_N_3_O_6_ | -0.68 | 1.26 | No | 4.6E-03 | 2.3E-02 | 0.54 |
| **Biogenic amines** |  |  |  |  |  |  |  |  |  |  |
| GABA | 25.7 | 104.0701 | 6.90 | C_4_H_9_NO_2_ | -0.3 | 0.7 | No | 1.4E-02 | 5.8E-02 | 0.27 |
| Tyramine | 3.2 | 138.0915 | 5.18 | C_8_H_11_NO | -0.5 | 0.9 | Yes | 6.4E-05 | 2.8E-03 | ^d^ |
| **Amino acids and derivatives** |  |  |  |  |  |  |  |  |  |  |
| 4-Hydroxyproline | 13.5 | 132.0650 | 7.97 | C_5_H_9_NO_3_ | 0.45 | 1.00 | Yes | 1.9E-01 | 3.2E-01 | 2.16 |
| *N*-Acetyl-2,4-diaminobutanoate/ Alal-Ala | 11.7 | 161.0946 | 5.72 | C_6_H_12_N_2_O_3_ | 0.65 | 1.36 | Yes | 1.8E-03 | 1.6E-02 | 5.38 |
| *N*_2_-Acetyl-ornithine/Theanine | 17.7 | 175.1068 | 5.86 | C_7_H_14_N_2_O_3_ | 0.44 | 1.02 | No | 1.7E-02 | 6.3E-02 | 1.90 |
| Ethyl glutamate/2-Aminoheptanedioic acid/hydroxyvalerylglycine | 12.1 | 176.0917 | 5.61 | C_7_H_13_NO_4_ | 0.47 | 0.92 | Yes | 3.8E-02 | 1.1E-01 | 4.06 |
| Ethyl glutamate/2-Aminoheptanedioic acid/hydroxyvalerylglycine | 4.5 | 176.0925 | 7.27 | C_7_H_13_NO_4_ | 0.42 | 1.00 | No | 1.6E-01 | 2.9E-01 | 2.14 |
| *N*-Hydroxy-phenylalanine/meta-tyrosine | 11.4 | 182.0809 | 7.62 | C_9_H_11_NO_3_ | -0.74 | 1.43 | No | 6.4E-05 | 2.8E-03 | ^d^ |
| 4-(Glutamylamino) butanoate/*N*_2_-Succinyl-ornithine/Aspartyl-Valine | 14.9 | 233.1133 | 6.36 | C_9_H_16_N_2_O_5_ | 0.71 | 1.33 | Yes | 2.0E-03 | 1.6E-02 | 5.70 |
| Cyclic argininosuccinic acid derivative 1 | 17.3 | 273.1189 | 5.72 | C_10_H_16_N_4_O_5_ | 0.65 | 1.24 | Yes | 8.7E-05 | 2.8E-03 | 50.82 |
| *N*_6_-(Octanoyl)lysine | 19.2 | 273.2158 | 6.15 | C_14_H_28_N_2_O_3_ | 0.68 | 1.38 | Yes | 4.1E-03 | 2.3E-02 | 3.27 |
| Proline betaine | 3.0 | 144.1030 | 5.00 | C_7_H_13_NO_2_ | 0.56 | 1.10 | Yes | 1.7E-02 | 6.3E-02 | 2.20 |

(^a^) Contains multiple identification options, (^b^) confidence intervals derived from jack-knife, 95 % confidence level, (^c^) FDR correction (*p*-values corrected by Benjamini Hochberg), (^d^) Only present in red wine group.

**Table S-6 cont.** List containing the statistically significant annotated metabolites discriminating between red and white wine profiles with their statistical characteristics after MVDA and UVDA.

|  |  |  |  |  | **MVDA** | | | **UVDA** | | **Fold change** |
| --- | --- | --- | --- | --- | --- | --- | --- | --- | --- | --- |
| **Compound Name** | **%RSD** | ***m/z*** | **MT**  **(min)** | **Formula** | ***p*(corr)** | **VIP** | **JK(^b^)** | ***p-*value** | **FDR(^c^)** |  |
| Pro-Ala | 12.7 | 187.1071 | 5.79 | C_8_H_14_N_2_O_3_ | 0.41 | 1.00 | No | 3.1E-02 | 1.0E-01 | 1.84 |
| Leu-Ala | 8.8 | 203.1380 | 6.02 | C_9_H_18_N_2_O_3_ | 0.55 | 1.23 | Yes | 7.6E-02 | 1.7E-01 | 2.49 |
| Thr-Ser | 19.9 | 207.0940 | 6.11 | C_7_H_14_N_2_O_5_ | 0.25 | 0.81 | No | 3.8E-02 | 1.1E-01 | 1.47 |
| Val Val | 4.7 | 217.1528 | 5.85 | C_10_H_20_N_2_O_3_ | 0.51 | 1.18 | Yes | 6.4E-02 | 1.5E-01 | 2.16 |
| Thr-Val | 7.3 | 219.1308 | 6.15 | C_9_H_18_N_2_O_4_ | 0.33 | 1.01 | No | 3.8E-01 | 5.3E-01 | 1.71 |
| Gly-Phe | 14.5 | 223.1088 | 6.09 | C_11_H_14_N_2_O_3_ | 0.70 | 1.37 | Yes | 3.4E-03 | 2.1E-02 | 5.10 |
| Ile-Val | 3.0 | 231.1695 | 6.17 | C_11_H_22_N_2_O_3_ | 0.37 | 1.02 | No | 2.1E-01 | 3.6E-01 | 1.75 |
| Ile-Ile | 8.4 | 245.1844 | 6.23 | C_12_H_24_N_2_O_3_ | 0.43 | 1.09 | No | 6.4E-02 | 1.5E-01 | 2.18 |
| Asp-Ile /Glu-Val | 12.4 | 247.1253 | 6.46 | C_10_H_18_N_2_O_5_ | 0.38 | 1.03 | No | 1.0E-01 | 2.1E-01 | 2.22 |
| Asp-Ile /Glu-Val | 19.1 | 247.1266 | 6.32 | C_10_H_18_N_2_O_5_ | 0.41 | 1.05 | No | 1.0E-01 | 2.1E-01 | 1.81 |
| Leu Lys | 6.9 | 260.1954 | 4.72 | C_12_H_25_N_3_O_3_ | 0.63 | 1.31 | Yes | 4.6E-03 | 2.3E-02 | 4.55 |
| Glu Leu | 9.9 | 261.1448 | 6.38 | C_11_H_20_N_2_O_5_ | 0.59 | 1.26 | Yes | 3.6E-03 | 2.1E-02 | 3.08 |
| Glu Lys | 8.6 | 276.1532 | 6.35 | C_11_H_21_N_3_O_5_ | 0.59 | 1.25 | Yes | 5.8E-03 | 2.8E-02 | 2.95 |
| Val Gly Leu | 14.0 | 288.1903 | 6.38 | C_13_H_25_N_3_O_4_ | 0.76 | 1.48 | Yes | 6.8E-04 | 9.2E-03 | 8.05 |
| Ile Arg | 18.1 | 288.2028 | 4.76 | C_12_H_25_N_5_O_3_ | 0.89 | 1.63 | Yes | 1.2E-04 | 2.9E-03 | 17.91 |
| Gly Thr Leu | 9.8 | 290.1701 | 6.40 | C_12_H_23_N_3_O_5_ | 0.69 | 1.38 | Yes | 1.2E-03 | 1.4E-02 | 5.34 |
| Leu Ala Val | 6.6 | 302.2053 | 6.44 | C_14_H_27_N_3_O_4_ | 0.66 | 1.34 | Yes | 1.3E-03 | 1.4E-02 | 4.88 |
| **Other compounds** |  |  |  |  |  |  |  |  |  |  |
| Picolinic acid/Nicotinic acid | 8.6 | 124.0394 | 7.11 | C_6_H_5_NO_2_ | -0.71 | 1.30 | Yes | 2.2E-03 | 1.6E-02 | 0.43 |
| Hypoxanthine |  | 137.0449 | 8.06 | C_5_H_4_N_4_O | -0.64 | 1.21 | No | 1.1E-02 | 5.1E-02 | 0.42 |
| 3-Dehydroxycarnitine | 13.6 | 146.1171 | 5.31 | C_7_H_15_NO_2_ | -0.55 | 1.08 | No | 4.5E-02 | 8.6E-02 | 0.59 |
| Imidazolelactic acid | 16.0 | 157.0602 | 5.65 | C_6_H_8_N_2_O_3_ | -0.50 | 0.92 | Yes | 2.6E-02 | 1.1E-01 | 2.05 |
| Ethyl N-ethylanthranilate | 8.9 | 194.1148 | 5.59 | C_11_H_15_NO_2_ | 0.62 | 1.23 | Yes | 2.6E-02 | 5.8E-02 | 0.43 |
| **Unknown** |  |  |  |  |  |  |  |  |  |  |
|  | 23.2 | 139.6059 | 4.54 |  | 0.84 | 1.57 | Yes | 3.1E-04 | 4.9E-03 | 10.88 |
|  | 17.6 | 160.6280 | 4.71 |  | 0.65 | 1.33 | Yes | 2.2E-03 | 1.6E-02 | 4.24 |

(^a^) Contains multiple identification options, (^b^) confidence intervals derived from jack-knife, 95 % confidence level, (^c^) FDR correction (*p*-values corrected by Benjamini Hochberg).
